# Supplementary material for: “I could not bear it”: Perceptions of chronic pain among Somali pastoralists in Ethiopia. A qualitative study
Source: PLoS One. 2023 Nov 13;18(11):e0293137. doi: 10.1371/journal.pone.0293137 (PMC10642812; doi:10.1371/journal.pone.0293137)
Supplement: S1 Appendix — (DOCX) [file pone.0293137.s001.docx]

**S1 Appendix**

Consolidated Criteria for Reporting Qualitative Research (COREQ)

| **No. Item** | **Manuscript section** | **Page number(s) in main manuscript** |
| --- | --- | --- |
| **Domain 1: Research Team and Reflexivity** | | |
| *Personal Characteristics* | | |
| 1. Interviewer/facilitator | Methods: Setting and recruitment Data collection, Context of the researchers | p. 6 - 9 |
| 1. Credentials | Title page; Methods: Context of the researchers | p. 9 |
| 1. Occupation | Title page; Methods: Context of the researchers | p. 9 |
| 1. Gender | Title page; Methods: Context of the researchers | p. 9 |
| 1. Experience and training | Methods: Data analysis, Context of the researchers | p. 8 & 9 |
| *Relationship with Participants* | | |
| 1. Relationship with participants | Methods: Context of the researchers | p. 9 |
| 1. Participant knowledge of the interviewer | Methods: Data collection; S2 Appendix | p. 7 & 8 |
| 1. Interviewer characteristics | Methods: Data analysis, Context of the researchers | p. 8 & 9 |
| **Domain 2: Study design** | | |
| *Theoretical framework* | | |
| 1. Methodological Orientation and Theory | Methods: Study design, Theoretical framework | p. 5 & 6 |
| *Participant Selection* |  |  |
| 1. Sampling | Methods: Sampling | p. 6 |
| 1. Method of approach | Methods: Sampling, Setting and recruitment | p. 6 & 7 |
| 1. Sample Size | Findings: Participant demographics and their pain conditions | p. 10 & 11 |
| 1. Non-participation | Not applicable | / |
| *Setting* | | |
| 1. Setting of data collection | Methods: Setting and recruitment | p. 6 & 7 |
| 1. Presence of non-participants | Strengths and limitations | p. 23 |
| 1. Description of sample | Findings: Participant demographics and their pain conditions | p. 10 - 12 |
| *Data Collection* | | |
| 1. Interview guide | Methods: Data collection; S2 Appendix | p. 7 & 8 |
| 1. Repeat interviews | Methods: Data collection, Trustworthiness; Strengths and limitations | p. 7 – 10, 23 |
| 1. Audio/visual recording | Methods: Data collection | p. 8 |
| 1. Field notes | Methods: Data collection, Data analysis, Trustworthiness | p. 8 & 9 |
| 1. Duration | Methods: Data collection | p. 7 |
| 1. Data saturation | Methods: Sampling; Strengths and limitations | p. 6, 23 |
| 1. Transcripts returned | Not Applicable | / |
| **Domain 3: Analysis and Findings** | | |
| *Data Analysis* | | |
| 1. Number of data coders | Methods: Data analysis | p. 8 & 9 |
| 1. Description of coding tree | Methods: Data analysis; Findings: Pastoralists’ perceptions of pain (Figure 1) | p. 8 & 9, p. 12 |
| 1. Derivation of themes | Methods: Data analysis; Findings | p. 8 & 9, 12 - 19 |
| 1. Software | Methods: Data analysis | p. 8 |
| 1. Participant checking | Methods: Trustworthiness; Strengths and limitations | p. 9 & 10, 23 |
| *Reporting* |  |  |
| 1. Quotations presented | Findings | p. 13 - 19 |
| 1. Data and findings consistent | Findings; Discussion; Conclusion | p. 10 – 25 |
| 1. Clarity of major themes | Findings (Figure 1) | p. 12 – 19 |
| 1. Clarity of minor themes | Findings (Figure 1) | p. 12 - 19 |
