# Supplementary material for: “I could not bear it”: Perceptions of chronic pain among Somali pastoralists in Ethiopia. A qualitative study
Source: PLoS One. 2023 Nov 13;18(11):e0293137. doi: 10.1371/journal.pone.0293137 (PMC10642812; doi:10.1371/journal.pone.0293137)
Supplement: S2 Appendix — (DOCX) [file pone.0293137.s002.docx]

**S2 Appendix**

Semi-structured interview guide

**Prior to starting interview**

- Introduction and information about the interviewer
- General Information about the study and purpose
- Ethical considerations, incl. informed consent

**Start of interview**

1^st^ Topic: Meaning of pain

- What does the word “pain” mean for you on a personal level?
- What do you associate with pain?
- How do you perceive pain in your society/in your community/in your family?

2^nd^ Topic: Personal pain perception

- What does your pain feel like to you?
- What words would you use to describe your pain?
- How do you show your pain to others?
- Why do you think you are suffering from pain?
- Where do you think the pain comes from?
- What started the pain?
- What makes the pain better or worse?
- How does your pain impact your everyday life or the life of your family?
- Imagine you would have nothing interfering with your wellbeing. Imagine you would have no pain and would be in perfect health. How would your daily life look like? (How) would it be different?

3^rd^ Topic: Dealing with pain

- What helps you to cope with pain?
- What do you do personally to reduce your pain?
- How do you deal with pain in your everyday life?
- Who do you consult when you are in pain or ask for help?
- What difficulties have you experienced in dealing with your pain?
- What could be done for you to improve the management of pain?
- What other kind of treatment would you like to receive?

**Closing remarks:**

- Is there anything you would like to talk about regarding this topic, that we have not mentioned yet?
- Expressing thanks
