# Supplementary material for: “I could not bear it”: Perceptions of chronic pain among Somali pastoralists in Ethiopia. A qualitative study
Source: PLoS One. 2023 Nov 13;18(11):e0293137. doi: 10.1371/journal.pone.0293137 (PMC10642812; doi:10.1371/journal.pone.0293137)
Supplement: Attachment — Submitted filename: Response to reviewers_.pdf [file pone.0293137.s003.pdf]

RESPONSE TO REVIEWERS

Manuscript: PONE-D-23-08854
Manuscript title: "I could not bear it": Perceptions of chronic pain among Somali pastoralists in Ethiopia. A qualitative study

**Comments from the Editor**	**Author's Response**	**Change in the Manuscript**
It would be nice if the sample size of the study could increased as suggested by the reviewers.	Thank you for your suggestion. We are convinced that qualitative studies with smaller sample sizes can provide valuable insights into a marginalized population that so far has been neglected in this field of research. We have now added more information on the sample size of the preceding focus group discussions in the methods section. We also mentioned this as a major strength of our study. Thereby, we can increase the overall sample size of the study.	- Methods section, p. 7, lines 181-186<br>- Strengths and limitations section, p. 23, lines 572-575
**Comments from Reviewer 1**		
Although, this study aims to explore the perceptions and notions of chronic pain among Somali pastoralists but I think this illustration cannot add more to the scientific research and the authors better use a control group from other country or another population to strenghthen the study.	Thank you for this comment. We argue that qualitative research has a great deal to offer when trying to gain in-depth insights into persons' illness experiences. From our point of view, the findings are completely novel in this field of research. To our knowledge no other study has examined chronic pain experience in this unique context and in this marginalized population. Indeed, these initial findings can provide valuable insights and orientation for future studies (as described in the "strengths and limitations"). In addition, by drawing on studies investigating Somalis and/or pastoralists in other countries, we can make relevant comparisons	- Methods section, p. 7, lines 181-186<br>- Strengths and limitations, p. 23, lines 570-575/590-593

	(e.g., lines 483-493). Furthermore, we made sure to now point out that the various settings of this study also allowed for interesting comparisons (home as well as biomedical).	
**Comments from Reviewer 2**		
I reviewed a manuscript titled "Perceptions of Chronic Pain Among Somali Pastoralists in Ethiopia." This is a small descriptive study that looked at a diverse group of adult pastoralists and agro-pastoralists dealing with chronic pain in Ethiopia. The study used ethnographic research techniques. Pastoralists are a group often marginalized socially and geographically, known for enduring many hardships in their daily lives. When it comes to chronic pain, they mainly rely on traditional and spiritual health practices, with Western biomedical approaches being quite rare. The authors referenced a study by Kawza et al., which found that only 10% of pastoralists in Southern Ethiopia used biomedical healthcare facilities when they were sick.<br><br>Main strength of this study is how it enhances our understanding of the sociocultural aspects of pain perception within this isolated population.<br><br>However, a major weakness lies in the small sample size and the potential bias towards patients who use biomedical healthcare facilities.	Thank you for your considerations and for highlighting these important points. We added further information on the utilization of health services specifically for Somali pastoralists.<br>As elaborated briefly above, we are convinced that qualitative studies with small sample sizes can provide valuable insights into a population, for which we have very limited information on pain perception. The small sample size was mentioned in the "strengths and limitations" section of the paper. We have now added more information on the sample size of the preceding focus group discussions. Indeed, most interviews took place in biomedical healthcare facilities. However, we also interviewed n=5 pastoralists affected by chronic pain in their home. We elaborate on this in the section "Setting and recruitment". In addition, we highlight the pastoralists' care itineraries in the results, thereby describing steps taken before they came to the health facility. In the "Conclusion", we added the recommendation to investigate how pastoralists are burdened by chronic pain and how they cope with chronic pain within their communities.	- Background section, p. 4, lines 88-94/99-103<br>- Methods section, p.7, lines 181-186<br>- Results section, p. 10, lines 268-269<br>- Strengths and limitations, p. 23, lines 572-575/591-593<br>- Conclusion section, p. 24, lines 598-600
